# Evaluation of electronic patient–reported outcome assessment in inpatient cancer care: a feasibility study

**DOI:** 10.1007/s00520-023-08014-9

**Published:** 2023-09-14

**Authors:** Hanna Salm, Leopold Hentschel, Martin Eichler, Daniel Pink, Stephan Fuhrmann, Michael Kramer, Peter Reichardt, Markus K. Schuler

**Affiliations:** 1 Department of Internal Medicine C, University Hospital Greifswald, Greifswald, Germany; 2 Sarcoma Center Berlin-Brandenburg, Helios Hospital Bad Saarow, Bad Saarow, Germany; 3 National Center for Tumor Diseases (NCT/UCC), Dresden, Germany; 4 Clinic for Hematology and Oncology, Helios Hospital Emil von Behring Berlin, Berlin, Germany; 5 AvenCell Europe GmbH, Dresden, Germany; 6 Clinic for Oncology and Palliative Medicine, Helios Hospital Berlin Buch, Berlin, Germany; 7 Division of Hematology, Oncology and Stem Cell Transplantation, Medical Clinic I, Department of Medicine I, University Hospital Carl Gustav Carus, Technische Universität Dresden, Fetscherstraße 74, 01307 Dresden, Germany; 8 Onkologischer Schwerpunkt am Oskar-Helene-Heim, Berlin, Germany

**Keywords:** Electronic assessment, Inpatient cancer care, Patient-reported outcomes, Feasibility

## Abstract

**Purpose:**

Patient-reported outcome (PRO) measures are increasingly important in evaluating medical care. The increased integration of technology within the healthcare systems allows for collection of PROs electronically. The objectives of this study were to Ashley et al. J Med Internet Res (2013) implement an electronic assessment of PROs in inpatient cancer care and test its feasibility for patients and Dawson et al. BMJ (2010) determine the equivalence of the paper and electronic assessment.

**Methods:**

We analyzed two arms from a study that was originally designed to be an interventional, three-arm, and multicenter inpatient trial. A self-administered questionnaire based on validated PRO-measures was applied and completed at admission, 1 week after, and at discharge. For this analysis — focusing on feasibility of the electronic assessment — the following groups will be considered: Group A (intervention arm) received a tablet version, while group B (control arm) completed the questionnaire on paper. A feasibility questionnaire, that was adapted from Ashley et al. J Med Internet Res (2013), was administered to group A.

**Results:**

We analyzed 103 patients that were recruited in oncology wards. ePRO was feasible to most patients, with 84% preferring the electronic over paper-based assessment. The feasibility questionnaire contained questions that were answered on a scale ranging from “1” (illustrating non achievement) to “5” (illustrating achieving goal). The majority (mean 4.24, SD .99) reported no difficulties handling the electronic tool and found it relatively easy finding time for filling out the questionnaire (mean 4.15, SD 1.05). There were no significant differences between the paper and the electronic assessment regarding the PROs.

**Conclusion:**

Results indicate that electronic PRO assessment in inpatient cancer care is feasible.

**Supplementary Information:**

The online version contains supplementary material available at 10.1007/s00520-023-08014-9.

## Introduction

The importance of the patients’ perspective on their well-being is increasingly recognized. Patient-reported outcome (PRO) measures are a valid method to provide valuable insight into patients’ experiences. PROs usually ask patients to self-report general well-being, symptoms, and functional status [2] revealing a patient-centered view on their subjective experiences [3].

By integrating PROs in the clinical routine, communication and engagement between healthcare providers and their patients is facilitated [4, 5]. If physicians have access to PROs, they can identify a higher number of symptoms [6]. In addition, PROs have shown to allow for more efficient use of clinic time [7]. Overall, the integration of PROs has resulted in better treatment quality [8, 9] and patients’ compliance [10]. Consequently, the collection and use of patient-reported information are extremely valuable.

PROs have been increasingly implemented in clinical practice as well as in research [11]. Many PRO measures have originally been completed on paper. However, there are several issues associated with paper versions of PROs. Frequent problems arise from incomplete questionnaires, making it difficult for healthcare professionals to accurately use data [12]. In addition, errors can occur due to manual scoring and data entry [13].

Subsequently, PRO measures have been altered to allow for electronic administration [14] offering a replacement to the traditional use of paper by using, for example, computers, tablets, or smartphones.

Studies have shown that electronic data capture offers several advantages: It reduces the number of data entry errors [15, 16] as well as the amount of missing data [17, 18]. Data are automatically calculated and transferred to a central database, strengthening the accuracy and efficiency of data collection [19]. Furthermore, the end user has immediate access to the data through the centralized database.

In addition, data capture can be improved since respondents can neither create their own response option nor have the opportunity to provide ambiguous responses [15, 17]. However, patients with less technical experiences may encounter difficulties when operating technical devices, such as tablets that were used in this study.

As the quality of data collection has improved and the use of data has become easier through electronic assessment, health care teams increasingly prefer electronic data capture over paper [20].

This has resulted in higher demand for research on the equivalence between electronic and paper-based PRO measures [14]. To target support and understand which mode of PRO-administration is most useful, we need to understand patients experience with the electronic assessment.

The purpose of this study was to [1] implement an electronic assessment of PROs in routine inpatient cancer care and test its feasibility. Furthermore, [2] we assessed whether the implementation of an electronic version of the standardized questionnaires results in equivalent responses to the paper version. In addition, [3] we examined the completion rate between paper-based and electronic assessment.

## Methods

### Ethical approval

The study was conducted in accordance with the Declaration of Helsinki and was approved by the Ethics Committee of the Chamber of Physicians in Berlin, Germany (Eth-48/16). All patients provided written informed consent to participate in the study.

### Study design

We evaluated the feasibility of an electronic PRO assessment tool in inpatient oncology care by conducting a multicenter, randomized, controlled trail. Patients were recruited between July 2017 and February 2019 while being admitted to four participating oncology wards (Helios Klinikum Emil von Behring, two centers at Helios Klinikum Berlin Buch, Helios Klinikum Bad Saarow) for planned anticancer treatment including chemotherapy, radiotherapy, or immunotherapy.

For the primary analysis, we randomized patients into three groups. As the current paper reports a secondary analysis, we analyzed two subgroups of the study: group A (now designated as the intervention arm) received a tablet version, while group B (control arm) completed the questionnaire on paper. PRO results from the third arm were graphically displayed and presented to their treating physicians for them to explore before their next patient encounter. However, this will be discussed in a further paper. Randomization was carried out using computerized routine by a staff member not further involved in the study.

Both groups received a set of standardized PRO questionnaires. While group B was given a paper version, group A completed the PROs in an electronic survey using an Apple iPad. Participants completed measurements independently but were allowed to ask for assistance.

There were three different points of measurement: T0: admission, T1: 1 week after admission (if applicable), and T2: discharge. Following T2, a feasibility questionnaire was administered to arm A to assess their experience using the electronic tool. If patients remained in the hospital less than 1 week, T1 did not take place.

A summary of the study design is depicted in the following consort diagram.

### Questionnaires

**Fig. 1:**
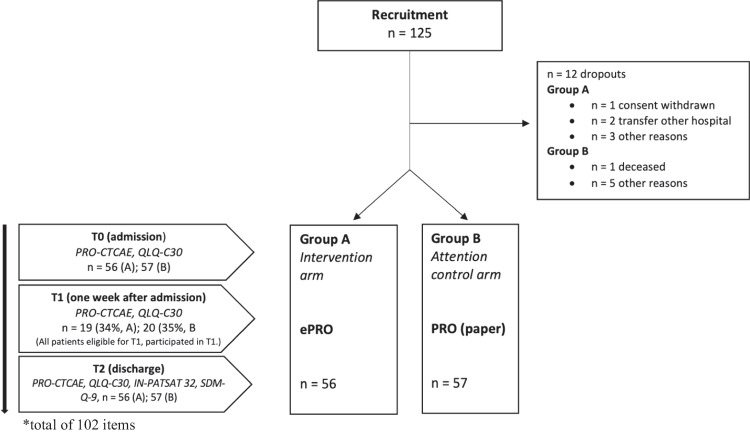
Consort diagram

The composition of PROs used for the study was developed in a multi-professional expert team and consisted of different instruments used at different assessment points (see Fig. 1). Standardized questionnaires with a total amount of 102 items were applied.

*EORTC QLQ-C30.* The Quality of Life Questionnaire C30 (QLQ-C30) [21] developed by the European Organization for Research and Treatment of Cancer (EORTC) assesses the global health status, five functional scales (physical, role, emotional, cognitive, social), and nine common symptoms in cancer patients. The QLQ-C30 consists of 30 items that relate to the state of health and well-being with scores ranging from 0 to 100. Higher symptom scores indicate higher symptom burden; however, higher scores in the global health status and the functional scales imply a better functioning.

*IN-PATSAT32.* The EORTC cancer inpatient satisfaction with care measure (IN-PATSAT 32) [22] holds 32-items assessing patients’ satisfaction with care by physicians and nurses. Higher values indicate greater satisfaction in the respective area.

*SDM-Q-9.* The German translation of the Shared Decision-making Questionnaire (SDM-Q-9) [23] assesses the extent of participatory decision-making*.* Patients indicate on a scale how appropriate individual elements of participation were at their last doctor’s visit. Higher values indicate a higher degree of participation.

*PRO-CTCAE.* Patient-reported adverse events are measured using the Common Terminology Criteria for Adverse Events (CTCAE) [24] that has also been adapted for patients to self-report (PRO-CTCAE) [25]. Patients rate the frequency, severity, and impairment of symptoms. For the present work, we only evaluated the severity. Modules were created that are suitable for the patients’ entities and treatments.

*Feasibility questionnaire* (shown in supplement). Feasibility is assessing the patients’ acceptability for the electronic assessment. The feasibility questionnaire presents 10 questions that were adapted from Ashley et al. [1] and applying goal attainment scaling [26].

Questions were answered on a scale ranging from “1” (illustrating non achievement) to “5” (illustrating achieving goal). Additionally, there were three items being answered with “yes” or “no”: “Did a staff member help you how to use the questionnaire today?,” “Did you need help from a staff member while answering the questions?,” and “Would you have preferred to answer the questions with pencil and paper?.” The feasibility questionnaire was administered to the intervention group (group A).

### Participants

Patients (aged 18 years or older) diagnosed with hematological or oncological cancer were eligible to enter the study. Hematological cancers relate to malignant hematological neoplasm, while we categorized any solid tumors as oncological. Eligibility was restricted to patients with a planned hospital stay for ≥ 3 days to undergo anticancer therapy.

### Statistical analysis

Scales of questionnaires were calculated according to the respective scoring manuals. For categorical variables, absolute frequencies and percentage are presented. For comparisons of the distributions of categorical variables between groups, the chi-squared-test [27] or Fisher’s exact test [28] (in case of counts of 5 or lower) were used. For continuous variables, arithmetic mean and standard deviation are presented. Continuous parameters between groups were compared using the Wilcoxon-Mann-Whitney-*U*-test [29, 30]. Overall significance level was 10% two-sided. Statistical analyses were conducted using SPSS version 27 and R version 4.0.1. Statistical analyses were pre-specified in a detailed statistical analysis plan.

## Results

Between July 2017 and February 2019, a total of *n* = 125 patients admitted for inpatient care were included in this study. Due to *n* = 12 dropouts (6 in group A, 6 in group B), the target sample resulted in *n* = 113 patients (100%). Patients were randomly assigned to two groups: group A consisted of 56 patients (100%) and group B of 57 patients (100%).

After 1 week, 34% of patients in group A and 35% in group B, that remained in the hospital participated in T1. The median duration of hospital stay was 6 days in group A and 7 days in group B. All patients included in the study participated at time of discharge (T2).

### Study population

Demographic data for patients is shown in Table 1.

**Table 1:** Characteristics of the study populations (*n* = 113)

Parameter	Group A (*n* = 56)	Group B (*n* = 57)	*p*
Age (years) (mean ± SD)	61.7 ± 12.5	65.7 ± 14.4	.04
Gender (*n*, %)			.18
Male	35 (62.5%)	28 (49.1%)	
Female	21 (37.5%)	29 (50.9%)	
BMI (kg/m^2^) (mean ± SD)	26.2 ± 6.4	25.1 ± 6.3	.38
Education (*n*, %)			.37
No vocational training	10 (17.9%)	14 (24.6%)	
Apprenticeship/university	46 (82.2%)	43 (75.4%)	
Missing/not applicable	0 (0%)	0 (0%)	
Employment before diagnosis (*n*, %)			.05
Employed	31 (55.4%)	26 (45.6%)	
Unemployed	3 (5.4%)	1 (1.8%)	
Retired	22 (39.3%)	29 (50.9%)	
Missing/not applicable	0 (0%)	1 (1.8%)	
Tumor type (*n*, %)			.69
Solid tumors	37 (66.1%)	35 (61.4%)	
Hematological malignancy	18 (32.1%)	21 (36.8%)	
Missing/not applicable	1 (1.8%)	1 (1.8%)	
Tumor stadium (*n*, %)			.19
I–III	16 (28.6%)	15 (26.3%)	
IV	16 (28.6%)	20 (35.1%)	
Missing/not applicable	24 (42.9%)	22 (38.6%)	

On average, patients in group A were 61.7 (SD ± 12.5) years and patients in group B were 65.7 (SD ± 14.4) years old. More than half in group A (62%, *n* = 35) and 49% (*n* = 28) in group B were male. The majority (82% in group A and 75% in group B) were educated with an apprenticeship or university degree. More than half in group A (55%) as well as 45% in group B were employed before diagnosis. Most patients (66% in group A and 61% in group B) were treated for oncological disease.

Stage IV tumor stadium applied to 28% in group A and 35% in group B.

### Completion rate

The following Table 2 shows completion rates for admission (T0), 1 week after admission (T1) and discharge (T2). We evaluated differences between the surveys as well as among the questionnaires.

**Table 2:** Completion rates

EORTC QLQ-C30	Group A			Group B		
	T0 (%)	T1 (%)	T2 (%)	T0 (%)	T1 (%)	T2 (%)
No entries (0%)	0.0	47.4	0.0	0.0	0.0	0.0
1–50% complete	0.0	0.0	0.0	0.0	0.0	0.0
51–90% complete	0.0	0.0	1.8	0.0	5.0	0.0
91–95% complete	5.4	5.3	3.6	1.8	10.0	5.3
96–99% complete	30.4	10.5	35.7	5.3	5.0	12.3
100% complete	64.3	36.8	58.9	93.0	80.0	82.5
PRO-CTCAE	Group A			Group B		
	T0 (%)	T1 (%)	T2 (%)	T0 (%)	T1 (%)	T2 (%)
No entries (0%)	1.8	5.3	0.0	0.0	0.0	0.0
1–50% complete	0.0	0.0	0.0	1.8	0.0	0.0
51–90% complete	1.8	0.0	1.8	0.0	0.0	1.8
91–95% complete	1.8	5.3	0.0	1.8	0.0	3.5
96–99% complete	1.8	0.0	1.8	3.5	0.0	3.5
100% complete	92.9	89.5	96.4	93.0	100.0	91.2
SDM-Q9	Group A			Group B		
	T0 (%)	T1 (%)	T2 (%)	T0 (%)	T1 (%)	T2 (%)
No entries (0%)	-	-	0.0	-	-	1.8
1–50% complete	-	-	3.6	-	-	1.8
51–90% complete	-	-	17.9	-	-	19.3
91–95% complete	-	-	0.0	-	-	0.0
96–99% complete	-	-	0.0	-	-	0.0
100% complete	-	-	78.6	-	-	77.2
IN PATSAT32	Group A			Group B		
	T0 (%)	T1 (%)	T2 (%)	T0 (%)	T1 (%)	T2 (%)
No entries (0%)	-	-	0.0	-	-	1.8
1–50% complete	-	-	3.6	-	-	1.8
51–90% complete	-	-	17.9	-	-	19.3
91–95% complete	-	-	0.0	-	-	0.0
96–99% complete	-	-	0.0	-	-	0.0
100% complete	-	-	78.6	-	-	77.2

For the PRO-CTCAE and the SDM-Q-9, completion rates at T2 were higher with the electronic assessment versus paper-based. However, the QLQ-C30 showed better completion rates for paper-based assessment at all assessment points. Significant differences between ePRO and paper-based assessment could be found: 64% at T0 (group A) versus 93% at T0 (group B), chi-squared test, *p* = 0.0005. As well as 59% at T2 (group A) versus 82% at T2 (group B), chi-squared-test, *p* = 0.011.

There was no difference regarding the 100% completion rate for the IN PATSAT32.

For the PRO-CTCAE, the completion rate was higher at admission (T0) than at discharge (T2).

The PRO-CTCAE was more often fully completed than the other questionnaires.

### Differences in PROs at T0 and T2

Results regarding QoL and symptom burden at admission (T0) are reported in Tables 3 and 4. High concordances were noted between the paper and the electronic version. Throughout all items, we did not observe significant differences between paper and electronic assessment of PROs. This indicates that electronic and paper-based assessment collect comparable information.

**Table 3:** Quality of life at admission (T0) and at discharge (T2)

	T0			T2		
EORTC QLQ-C30	Group A score (SD)	Group B score (SD)	*p*	Group A score (SD)	Group B score (SD)	*p*
Global health status	56 (21)	54 (26)	.57	50 (19)	50 (22)	.94
Physical functioning	31 (25)	36 (26)	.39	37 (28)	40 (28)	.47
Role functioning	53 (34)	48 (32)	.37	52 (39)	48 (35)	.57
Emotional functioning	40 (27)	38 (23)	.74	36 (29)	30 (24)	.36
Cognitive functioning	18 (20)	24 (24)	.15	21 (21)	23 (22)	.60
Social functioning	48 (33)	46 (33)	.66	48 (32)	43 (32)	.43
Fatigue	51 (28)	51 (26)	.98	56 (29)	52 (28)	.42
Nausea and vomiting	10 (18)	11 (20)	.55	21 (27)	20 (29)	.52
Pain	35 (34)	33 (32)	.86	34 (31)	25 (29)	.08
Dyspnea	32 (32)	29 (32)	.47	31 (32)	23 (30)	.15
Insomnia	35 (31)	39 (35)	.73	39 (32)	38 (35)	.82
Appetite loss	31 (38)	32 (39)	.94	51 (38)	43 (41)	.25
Constipation	21 (27)	22 (35)	.52	26 (29)	22 (33)	.29
Diarrhea	15 (25)	16 (26)	.68	22 (31)	20 (30)	.57
Financial difficulties	29 (36)	20 (30)	.27	31 (38)	23 (34)	.26

Calculated score (range 1–100). Function score high = good function. Symptom score high = high symptom burden

**Table 4:** Symptom burden at (T0) and at discharge (T2)

	T0			T2		
PRO-CTCAE	Group A score (SD)	Group B score (SD)	*p*	Group A score (SD)	Group B score (SD)	*p*
Nausea	.65 (1.2)	.63 (.96)	.73	1.1 (1.3)	1.1 (1.3)	.88
Vomiting	.27 (.88)	.20 (.71)	.70	.56 (1.2)	5.9 (1.1)	.86
Pain	1.3 (1.2)	1.3 (1.2)	.96	1.3 (1.2)	1.0 (1.2)	.20
Constipation	.58 (1.1)	.89 (1.2)	.13	.80 (1.1)	.74 (1.1)	.44
Decreased appetite	1.1 (1.5)	1.1 (1.3)	.79	1.8 (1.3)	1.5 (1.4)	.23
Difficulty swallowing	.33 (.85)	.23 (.60)	.80	.66 (1.1)	.46 (.98)	.25
Dry mouth	.95 (1.0)	1.1 (1.2)	.64	1.4 (1.1)	1.1 (1.1)	.27
Mouth/throat scores	.13 (.34)	.23 (.63)	.72	.30 (.76)	.33 (.79)	.83
Numbness and tingling	.95 (1.1)	.91 (1.2)	.79	.88 (1.1)	1.0 (1.1)	.32
Shortness of breath	.80 (1.0)	.98 (1.2)	.60	.93 (1.1)	.68 (.92)	.28
Fatigue	1.6 (1.2)	1.8 (1.1)	.18	1.8 (1.2)	1.8 (1.2)	.78
Concentration	.70 (.84)	.93 (1.0)	.34	.88 (.93)	1.0 (1.1)	.70
Insomnia	1.3 (1.2)	1.6 (1.2)	.33	1.4 (1.1)	1.5 (1.3)	.86
Anxious	.98 (1.2)	.96 (1.3)	.87	.98 (1.2)	.70 (1.1)	.10
Sad severity	1.2 (1.2)	1.4 (1.2)	.46	1.1 (1.1)	.93 (1.0)	.27

Calculated score (range 0–4). Symptom score high = high symptom burden

### Feasibility

Feasibility results are reported in Table 5. Almost 79% participants reported not needing support for answering the questionnaire indicating that the electronic assessment was broadly acceptable for participants. However, 51.8% required help to operate the questionnaire.

**Table 5:** Feasibility (*n* = 56)

Parameter	Group A
	T2
Required help to operate the questionnaire *n* (%)	56 (100%)
Yes	29 (51.8%)
No	26 (46.4%)
Missing/not applicable	1 (1.8%)
Received support for answering the questions *n* (%)	56 (100%)
Yes	11 (19.6%)
No	44 (78.6%)
Missing/not applicable	1 (1.8%)
Satisfaction with help from staff (mean ± SD)	3.75 ± 1.31
*N*; Missing/not applicable	44; 12
Difficulty finding time answering the questionnaire (mean ± SD)	4.15 ± 1.05
*N*; Missing/not applicable	54; 2
Satisfaction with number of questions asked *n* (%)	56 (100%)
Would have answered more	3 (5.4%)
Adequate	33 (58.9%)
Too many	18 (32.1%)
Missing/not applicable	2 (3.6%)
Difficulty operating the questionnaire (mean ± SD)	4.24 ± 0.99
*N*; Missing/not applicable	54; 2
Satisfaction regarding filling out the questionnaire (mean ± SD)	3.78 ± 0.88
*N*; Missing/not applicable	54; 2
Willingness to continue answering more questions with the system (mean ± SD)	3.59 ± 1.60
*N*; Missing/not applicable	54; 2
Prefer using pen and paper over electronic questionnaire *n* (%)	56 (100%)
Yes	6 (10.7%)
No	47 (83.9%)
Missing/not applicable	3 (5.4%)

Respondents needing help presented a relatively high satisfaction with the support they received (mean 3.75, SD 1.31). This question was added to all patients, even to those not actively asking for help. We did assume our staff to give an introduction in the study rationale, procedure, and device handling. High satisfaction with help can therefore also be interpreted with the staff supporting our patients satisfactorily if any assistance was needed. Furthermore, the patients commonly reported that they found it relatively easy finding time for filling out the questionnaire (mean 4.15, SD 1.05).

The majority (mean 4.24, SD .99) reported no difficulties handling the electronic assessment.

Patients were generally satisfied with the completion of the questionnaire (mean 3.78, SD .88). More than 59% participants found the number of questions asked to be adequate and the majority would have continued answering more questions with the system (mean 3.59, SD 1.60).

The majority (84%) would have not preferred to complete the questionnaires in a paper version indicating a preference for the electronic assessment.

## Discussion

As previously discussed, self-assessment and external assessment of patients’ symptoms tend to diverge [31–33] and patients’ subjective view can never be reliably represented except from the patient itself. PROs and their efficient assessment are therefore of major importance.

However, as previously suggested [34], incorporating ePRO measures into existing workloads was a significant barrier.

### Feasibility

We found that the electronic tool was feasible as most patients reported no difficulties handling the electronic assessment indicating that the tool was broadly acceptable for patients.

However, there was a difference between operating the questionnaire and answering the questions itself. While 78.6% reported not needing help for answering the questions, more than half (51.8%) did require help to operate the questionnaire. Possibly, this is related to the age of the patients, as older age is associated with lower computer and internet use [35]. In the long term, the internet will become established almost universally. We still consider the tool feasible as the ePRO assessment was well received with the majority presenting a relatively high satisfaction with the tool. Furthermore, patients would not prefer to complete the questionnaires in a paper version, which indicates a preference for the electronic assessment.

Those findings suggest good feasibility, suggesting that the electronic capture of PROs provides a reliable replacement for the paper form. This is consistent with other research findings comparing electronic and paper assessment of PROs [17, 18] and showing good concordance between the surveys across a wide variety of diseases [36].

Multiple benefits are associated with ePRO implementation. If ePROs were implemented in routine care, data could be efficiently stored in one location and was immediately available for the healthcare professionals to review in the data base. In addition to these improvements, our results have shown that patients are satisfied with the electronic capture. This is consistent with previous studies comparing electronic forms to paper forms [37–40]. As already stated, our patients showed little problems handling the electronic tool which aligns with previous studies that suggest high acceptability among patients using tablet based assessment tools [41, 42].

In addition, electronic completion may be easier for patients with limited manual dexterity [43].

Electronic surveys are also perceived as more anonymous than paper-based surveys [44], potentially leading to greater honesty on the patients side.

While there are advantages being associated with electronic assessment of PROs, it should be kept in mind that studies, that require an electronic device, risk excluding patients whose insight and experiences with technology are limited.

### Completion rate

We included four questionnaires with a total of 102 items in the study. For the PRO-CTCAE and the SDM-Q-9, completion rates at T2 were higher with the electronic assessment versus paper-based. However, the QLQ-C30 showed better completion rates for paper-based assessment at all assessment points. This shows that neither the electronic nor the paper-based assessment consistently resulted in better completion rates.

For the QLQ-C30, nearly half of those in group A (47%) had no entries in the questionnaire at T1. This is quite surprising, especially since at all other timepoints a minimum of 85% patients in both groups reached at least 96% completion rate. We were not able to completely clarify the reason for this deviation. Most likely it was due to a technical issue based on the study staff not appropriately administering the survey. This shows that even with electronic administration, manual input can still be necessary if problems occur.

Overall, a high number of questionnaires were not fully completed. This leads to the question whether an unsuitable amount of items was chosen for this study. Considering the fact that patients undertook the survey more than once, it is thinkable that the number of items was too high. This can be underlined by a previous study that indicated a negative correlation between the completion rate and survey length [45]. The burden of symptoms could also have an impact on questionnaire completion, as described in further studies [46, 47]. If patients are unwell, this might lead to difficulties completing PROs, regardless if they are electronic or paper based.

If PROs are seen to be useful for clinical care, then patients are more likely to overcome barriers to achieve completion.

### Limitations

There were some limitations that should be considered when interpreting these findings. The main limitation of this study was that the feasibility questionnaire was only given to the intervention group that used the electronic assessment. Therefore, we cannot report on patients’ experiences with the paper form.

We also assessed differences within the PROs. However, the questionnaires were completed only either in the electronic or the paper version. Respondents were not exposed to both surveys, making direct comparison somewhat difficult.

The relatively short median duration of hospitalization (6 days in group A and 7 days in group B) could have affected the survey completion rates. A longer stay, so that all patients could have participated in T1, would have increased the informative value about differences between electronic and paper-based assessment.

## Conclusion

Our study concludes that the assessment of electronic PRO measures is feasible in routine inpatient cancer care. The majority (84%) would have not preferred to complete the questionnaires in a paper version indicating a preference for the electronic assessment. Patients were therefore receptive to the electronic assessment and showed little problems handling the tool. Our findings support that electronic assessment is a valuable replacement to paper versions. The results of this study can allow for better understanding of the complexity of ePRO implementation and be helpful in creating strategies for further application.

### Supplementary information

ESM 1 (DOCX 75 kb)
