# Supplementary material for: Evaluation of electronic patient–reported outcome assessment in inpatient cancer care: a feasibility study
Source: Support Care Cancer. 2023 Sep 14;31(10):575. doi: 10.1007/s00520-023-08014-9 (PMC10501936; doi:10.1007/s00520-023-08014-9)
Supplement: Supplementary file 1 — (DOCX 75 kb) [file 520_2023_8014_MOESM1_ESM.docx]

**Supplement**

Feasibility questionnaire

|  |
| --- |
| 1) What kind of device did you use to answer the questions of this study with? |
| □ Computer at home  □ Computer at a relative’s or friend’s home  □ Computer at the clinic  □ Computer in a public place (e.g., library or Internet café)  □ On a tablet (e.g., iPad)  □ On a mobile phone |
| 2) Did a study staff member help you operating the questionnaire today? |
| Yes □ No □ |
| 3) Did you need assistance from a study staff member while answering the questions? |
| Yes □ No □ |
| 4) How satisfied were you with the support you received? |
| □ 5 – Very satisfied  □ 4  □ 3  □ 2  □ 1- Very unsatisfied |
| 5) How difficult did you find it to find time to answer the questions? |
| □ 5 – Very easy  □ 4  □ 3  □ 2  □ 1 – Very difficult |
| 6) How did you feel about the number of questions asked? |
| □ I would have answered more □ Adequate number □ Too many |
| 7) How easy was it for you to use the questionnaire? |
| □ 5 – Very easy  □ 4  □ 3  □ 2  □ 1 – Very difficult |
| 8) How satisfied were you with the complication of the questionnaire? |
| □ 5 – Very satisfied  □ 4  □ 3  □ 2  □ 1 – Very unsatisfied |
| 9) If you were asked: Would you be willing to answer more questions with the electronic system? |
| □ 5 – Very likely  □ 4  □ 3  □ 2  □ 1 – Very unlikely |
| 10) Would you have prepared to answer the questions with paper and pencil? |
| Yes □ No □ |
